# Metabolic syndrome and its component traits present gender-specific association with liver cancer risk: a prospective cohort study

**DOI:** 10.1186/s12885-021-08760-1

**Published:** 2021-10-07

**Authors:** Bin Xia, Jianjun Peng, De Toni Enrico, Kuiqing Lu, Eddie C. Cheung, Zichong Kuo, Qiangsheng He, Yan Tang, Anran Liu, Die Fan, Changhua Zhang, Yulong He, Yihang Pan, Jinqiu Yuan

**Affiliations:** 1 Clinical Research Center, The Seventh Affiliated Hospital, Sun Yat-sen University, Shenzhen, 518107 Guangdong China; 2 Big Data Centre, The Seventh Affiliated Hospital, Sun Yat-sen University, Shenzhen, 518107 Guangdong China; 3 Guangdong Provincial Key Laboratory of Gastroenterology, Center for Digestive Disease, The Seventh Affiliated Hospital, Sun Yat-sen University, Shenzhen, Guangdong China; 4 Department of Gastrointestinal Surgery, The First Affiliated Hospital of Sun Yat-sen University, Guangzhou, Guangdong China; 5 Department of Medicine II, University Hospital, LMU Munich, Munich, Germany; 6 Division of Gastroenterology, Davis School of Medicine, University of California, Oakland, USA; 7 Department of Clinical Nutrition, The Seventh Affiliated Hospital, Sun Yat-sen University, Shenzhen, Guangdong China; 8 Precision Medicine Center, Scientific Research Center, The Seventh Affiliated Hospital, Sun Yat-sen University, Shenzhen, Guangdong China

**Keywords:** Metabolic syndrome, Liver cancer, Gender-specific effect, Non-linear association, UK Biobank

## Abstract

**Background & Aims:**

Little is known on the gender-specific effect and potential role of non-linear associations between metabolic syndrome (MetS) components and liver cancer risk. We evaluated these associations based on the UK Biobank cohort.

**Methods:**

We included 474,929 individuals without previous cancer based on the UK Biobank cohort. Gender-specific hazard ratios (HRs) and 95% confidence interval (CIs) were calculated by Cox proportional hazards regression, adjusting for potential confounders. Non-linear associations for individual MetS components were assessed by the restricted cubic spline method.

**Results:**

Over a median follow-up of 6.6 years, we observed 276 cases of liver cancer (175 men, 101 women). MetS [HR 1.48, 95% CI 1.27–1.72] and central obesity [HR 1.65, 95% CI 1.18–2.31] were associated with higher risk of liver cancer in men but not in women. Participants with hyperglycaemia has higher risk of liver cancer. High waist circumference and blood glucose were dose-dependently associated with increased liver cancer risk in both genders. For high density lipoprotein (HDL) cholesterol (both genders) and blood pressure (women), U-shaped associations were observed. Low HDL cholesterol (< 1.35 mmol/L) in men and high HDL cholesterol in women (> 1.52 mmol/L) were associated with increased liver cancer risk.

**Conclusions:**

MetS components showed gender-specific linear or U- shaped associations with the risk of liver cancer. Our study might provide evidence for individualized management of MetS for preventing liver cancer.

**Supplementary Information:**

The online version contains supplementary material available at 10.1186/s12885-021-08760-1.

## Introduction

Liver cancer is the sixth most commonly diagnosed cancer and the third leading cause of cancer-related deaths worldwide, accounting for more than 0.91 million new cases and 0.83 million deaths per year [1]. Apart from chronic hepatitis B and C viral infections and excessive alcohol consumption, non-alcoholic fatty liver disease (NAFLD), a spectrum of liver pathology that resembles alcohol-induced fatty liver damage without other known causes of steatosis, has been identified as a major risk factor for liver cancer [2]. According to epidemiological studies, 4–22% of liver cancer cases in Western countries are attributed to NAFLD [2, 3].

NAFLD is a cause and a consequence of metabolic syndrome (MetS), an assemblage of metabolic abnormalities that relates to the occurrence of cardiovascular diseases and oncogenesis [3]. It has been shown that subjects with MetS tend to have an increased hepatic insulin resistance and fat (triglyceride) accumulation in the liver, which may increase the risk of NAFLD and non-alcoholic steatohepatitis (NASH) [4]. Although accumulating evidence suggests that MetS might play a role in hepatocarcinogenesis [5–8], the results were inconsistent, especially concerning the gender-specific effects of critical MetS components. For instance, many studies suggest that MetS [8], metabolic risk score [9], or a prolonged duration of diabetes [10], might increase the risks of hepatocellular carcinoma (HCC), especially in males, while others did not find such associations [9, 11, 12].

In addition, evidence concerning the hepatocarcinogenesis effect of low level of MetS components is increasing. In the study by Wei et al., waist circumference (WC) showed a U-shaped association with primary liver cancer (PLC) risk. Both males with high or low WC were associated with an increased risk of PLC [13]. This suggests the importance of controlling WC in an appropriate range, neither too high nor too low, in PLC primary prevention. However, the non-linear relationships concerning other MetS components have rarely been investigated. A further investigation of the gender-specific metabolic factors and their potential non-linear relationship with liver cancer may contribute to the individualized prevention. Thus, based on the UK Biobank dataset, we undertook this prospective analysis to 1) elucidate the relationship of MetS and metabolic factors including central obesity, hyperglycaemia, hypertension, dyslipidemia with risk of liver cancer with emphasis on sex disparity; 2) investigate the potential non-linear associations between metabolic factors and liver cancer risk.

## Materials and methods

### Data source and study design

This is a prospective study based on UK Biobank (application number 51671, approved August 2019), which is on-going cohort with over 500,000 individuals aged 40–69 years recruited throughout England, Wales, and Scotland between 2006 and 2010. Details on the study design and assessments of the UK Biobank cohort have been published elsewhere [14]. UK Biobank was approved by the National Information Governance Board for Health and Social Care in England and Wales, and the Community Health Index Advisory Group in Scotland. All participants had provided written informed consent prior to data collection.

The UK Biobank dataset included 502,527 participants. Participants were excluded if they had any cancer diagnosis prior to baseline (except for non-melanoma skin cancer ICD-10 C44) (*n* = 26,868), had missing data on measure of all five components of metabolic syndrome (*n* = 581), and were pregnant (*n* = 149). The participants were followed until the date of first liver cancer diagnosis or censoring (30 October 2015). A total of 474,929 participants (219,383 men and 256,276 women) were included in the final analysis (see supplementary file Figure S1).

### Data collection

Information on socio-demographic characteristics, lifestyle factors, comorbidities and medication for each participant were collected from patient interview or a touchscreen questionnaire at baseline. Diet intake was evaluated using the food frequency questionnaire, and physical activity was assessed using adapted questions from the validated short International Physical Activity Questionnaire (IPAQ). Anthropometric measurements of height, weight, waist circumference, and blood pressure were performed during the physical examination. Blood samples were obtained from each participant after a 12 h overnight fast and serum fasting blood glucose, HDL cholesterol, triglycerides were assessed using a Beckman Coulter AU5800 analyzer (Beckman Coulter (UK) Ltd., High Wycombe, United Kingdom).

### Definition for MetS and MetS components

Several organizations have developed the diagnostic criteria for MetS since 1998. In the present study, we defined MetS based on the most recent interim joint consensus definition incorporating International Diabetes Federation (IDF) and American Heart Association/National Heart, Lung and Blood Institute (AHA/NHLBI) criteria (the occurrence of any three or more components constitutes a diagnosis of MetS) [15]. According to the criteria, central obesity was defined based on WC with cutoff-points that are gender and ethnic-group specific. Hyperglycaemia was defined as fasting plasma glucose ≥5.56 mmol/L (100 mg/dL) or previously diagnosed type 2 diabetes. Raised blood pressure was defined as systolic blood pressure (SBP) ≥ 130 mmHg, diastolic blood pressure (DBP) ≥ 85 mmHg, or antihypertensive treatment for previously diagnosed hypertension. Elevated triglycerides was defined as plasma Triglycerides (TG) levels ≥1.7 mmol/L (150 mg/dL) or currently on medications for hypertriglyceridaemia. Low HDL cholesterol was defined as HDL < 1.03 mmol/L (40 mg/dL) for male and < 1.29 mmol/L (50 mg/dL) in female or specific treatment for previously detected reduced HDL cholesterol level.

### Ascertainment of cancer cases

Prevalent and incident liver cancer cases within the UK Biobank cohort were identified through linkage to cancer and death registries from the Health and Social Care Information Centre (in England and Wales) and the National Health Service Central Register (in Scotland). Eligible participants contributed person-years from recruitment date until date of the first liver cancer diagnosis (ICD-10 codes C22), date of death, or the last date of follow-up (30 October 2015), whichever came first.

### Statistical analysis

In descriptive analyses, values were presented as either a mean (standard deviation) or number (percentage). For covariates with selections of ‘do not know’ and ‘prefer not to answer’, or with missing covariate data, an “unknown/missing” response category was created.

Cox proportional hazard regression analyses, taking age as the time variable, were applied to evaluate the associations of MetS and its components with subsequent liver cancer risk. The effects were presented with hazards ratio (HR) and 95% confidence interval (95% CI). Proportional hazards assumption was checked using Schoenfeld’s tests. For the main analysis (model 1), the following covariates were included: age in years, ethnic, education, index of multiple deprivation (a measure of socio-economic status), alcohol consumption, smoking status, physical activity, portions of fruit and vegetable intake, comorbidities, family history of cancer, menopause status (for the female only) and hormone replacement therapy (for the female only). With low variance inflation factor (VIF) values of below 1.3, the five components of MetS were further included in the model 2 to test their independent association with subsequent liver cancer risk. To evaluate the continuous effect across MetS components (analyzed as continuous variables), we used restricted cubic spline with four knots at the 5th, 35th, 65th, and 95th centiles to flexibly model the potential non-linearity association of each MetS component with risk of liver cancer. We investigated the non-linearity associations by using a likelihood ratio test comparing the model with only a linear term against the model with linear and cubic spline terms. Interaction analyses were conducted to investigate the diversity of risk of liver cancer by gender.

We performed several sensitivity analyses to check the robustness of the primary results. First, we evaluated the gender-specific risk of liver cancer in association with MetS defined according to the IDF criteria, AHA/NHLBI criteria and NCEP ATP III criteria. Second, the liver cancer cases occurred in the first 2 years of follow-up were excluded to reduce potential reverse causation. Third, we used the complete case analysis to verify the influence of missing data. Last, we excluded individuals with a history of hepatitis, liver failure and/or cirrhosis, which are the most common risk factors for liver cancer. All statistical analyses were performed using the R software (version 3.5.0, R Foundation for Statistical Computing, Vienna, Austria).

## Results

We documented 276 incident cases of liver cancer (175 men, 101 women) among 474,929 included participants (219,383 men and 256,276 women) over 3,116,398 person-years of follow-up. Table 1 represents the gender-specific baseline participant characteristics by MetS status. Nearly a quarter of participants were classified as with MetS (men: *N* = 56,766, 25.9%; women: *N* = 56,766, 22.6%). Participants with MetS tended to be older, have a higher multiple deprivation index, and more likely to be Caucasians. As expected, they also had higher BMI, waist circumference, blood pressure and serum concentrations of triglyceride, fasting glucose, and lower serum concentrations of HDL-cholesterol than the non-MetS group. More previous smokers, light drinkers, and individuals with lower physical activity or fruit/vegetable intake were observed in the MetS group than in the non-MetS group.

**Table 1:** Baseline characteristics of included participants

Characteristics	Males		Females	
	No MetS (*N* = 162,301)	MetS (*N* = 56,766)	No MetS (*N* = 198,275)	MetS (*N* = 57,736)
Mean (SD) age, years	56.1 (8.29)	57.8 (7.79)	55.5 (8.06)	58.3 (7.49)
White, N (%)	151,840 (93.6)	53,935 (95.0)	187,044 (94.3)	53,775 (93.1)
Mean (SD) IDM	17.5 (14.0)	19.6 (15.1)	16.7 (13.2)	20.1 (15.2)
Mean (SD) BMI, kg/m2	26.3 (3.12)	32.3 (3.80)	25.5 (4.11)	32.4 (5.00)
Mean (SD) WC, cm	92.6 (8.59)	109 (8.96)	80.5 (9.99)	98.8 (9.84)
Mean (SD) SBP, mmHg	141 (18.6)	147 (17.6)	135 (20.2)	145 (18.4)
Mean (SD) DBP, mmHg	83.1 (10.5)	86.9 (10.4)	79.4 (10.4)	85.1 (10.1)
Mean (SD) HDL cholesterol, mmol/L	1.34 (0.312)	1.12 (0.250)	1.67 (0.364)	1.34 (0.296)
Mean (SD) TG, mmol/L	1.76 (1.01)	2.57 (1.29)	1.33 (0.667)	2.23 (1.02)
Mean (SD) fasting glucose, mmol/L	5.03 (1.09)	5.62 (1.98)	4.93 (0.778)	5.47 (1.62)
**Smoking status, N (%)**				
Never smoked	83,860 (51.7)	23,289 (41.0)	120,211 (60.6)	32,336 (56.0)
Previous smoker	56,910 (35.1)	26,228 (46.2)	59,753 (30.1)	19,540 (33.8)
Current smoker	20,721 (12.8)	6879 (12.1)	17,442 (8.8)	5465 (9.5)
**Alcohol consumption, N (%)**				
Daily or almost daily	42,423 (26.1)	12,884 (22.7)	34,531 (17.4)	6477 (11.2)
Three or four times a week	43,741 (27.0)	13,430 (23.7)	44,056 (22.2)	8532 (14.8)
Once or twice a week	41,329 (25.5)	15,272 (26.9)	52,150 (26.3)	13,661 (23.7)
One to three times a month	13,721 (8.5)	5782 (10.2)	24,699 (12.5)	8679 (15.0)
Special occasions only	10,904 (6.7)	5155 (9.1)	26,118 (13.2)	12,258 (21.2)
Never	9764 (6.0)	4107 (7.2)	16,333 (8.2)	7958 (13.8)
**Physical activity, N (%)**				
Low	22,879 (14.1)	12,252 (21.6)	25,960 (13.1)	10,799 (18.7)
Moderate	51,856 (32.0)	18,397 (32.4)	66,656 (33.6)	18,210 (31.5)
High	61,953 (38.2)	16,130 (28.4)	63,128 (31.8)	13,066 (22.6)
**Portions of fruit and vegetable, N (%)**				
< 5 portions per day	110,871 (68.3)	39,150 (69.0)	110,931 (55.9)	33,850 (58.6)
≥ 5 portions per day	50,871 (31.3)	17,414 (30.7)	86,968 (43.9)	23,718 (41.1)
**Comorbidities**				
Hepatitis, N (%)	204 (0.1)	68 (0.1)	186 (0.1)	52 (0.1)
Liver failure/cirrhosis, N (%)	101 (0.1)	68 (0.1)	116 (0.1)	54 (0.1)

*Abbreviations*: *BMI* body mass index, *DBP* diastolic blood pressure, *HDL* high density lipoproteins, *IDM* index of multiple deprivation, *MetS* metabolic syndrome, *SBP* systolic blood pressure, *TG* triglycerides, *WC* waist circumference

Table 2 illustrated the gender-specific risk of liver cancer in association with MetS. The male participants with MetS had a 48% increased risk of liver cancer as compared with those without MetS (HR = 1.48, 95% CI, 1.27–1.72). We did not find sufficient evidence of association between MetS and risk of liver cancer in women. The results did not change substantially in the sensitive analysis of defining MetS according to IDF criteria, NCEP ATP III criteria or AHA/NHLBI criteria (see supplementary file Table S1). The results were generally unchanged in other sensitivity analyses by lagging the exposure for a time window of 2 years, applying the complete case analysis, or excluding individuals with a history of hepatitis, liver failure or cirrhosis (see supplementary file Table S2).

**Table 2:** Gender-specific hazard ratio and 95% confidence interval for liver cancer by metabolic syndrome

	Males		Females	
	No. of cases/ Person-years	HR (95% CI)^a^	No. of cases/ Person-years	HR (95% CI)^a^
Without MetS	54/786,106	Ref	67/1,135,661	Ref
With MetS	121/643,669	1.48 (1.27–1.72)^***^	34/550,962	1.00 (0.78–1.29)

*Abbreviations*: *CI* confidence interval, *HR* hazard ratio, *MetS* metabolic syndrome

^***^P-value < 0.001

^a^Estimates were assessed by multivariable adjusted Cox proportional hazards model adjusted for age in years, ethnic, education, index of multiple deprivation (a measure of socio-economic status), alcohol consumption, smoking status, physical activity, portions of fruit and vegetable intake, comorbidities, family history of cancer, menopause status (for the female only) and hormone replacement therapy (for the female only)

Table 3 presents the gender-specific effects of individual MetS components on liver cancer risk. For male participants, central obesity (HR = 1.62, 95% CI, 1.16–2.27) and hyperglycaemia (HR = 1.70, 95% CI, 1.22–2.59 for pre-diabetes, HR = 2.88, 95% CI, 1.91–4.34 for diabetes) showed increased hazard for liver cancer in the fully-adjusted model. Female participants with diabetes also present association with increased hazard for liver cancer (HR = 2.43, 95% CI, 1.20–4.91). We did not observe sufficient evidence of associations of other MetS components with liver cancer risk in women.

**Table 3:** Gender-specific hazard ratio and 95% confidence interval for liver cancer risk by metabolic syndrome components

	Males			Females		
	No. of cases/ Person-years	HR (95% CI)		No. of cases/ Person-years	HR (95% CI)	
		Model 1^a^	Model 2^b^		Model 1^a^	Model 2^b^
**Central obesity**						
No	40/585,033	Ref	Ref	36/658,830	Ref	Ref
Yes	135/844,742	1.87 (1.30–2.67)^***^	1.62 (1.16–2.27)^**^	65/1,027,793	0.85 (0.56–1.30)	1.05 (0.66–1.68)
**Hypertension**						
No	11/275,537	Ref	Ref	36/571,100	Ref	Ref
Yes	164/1,151,248	1.31 (0.86–1.98)	1.16 (0.75–1.79)	64/1,111,874	0.63 (0.41–0.95)^*^	0.71 (0.45–1.13)
**Elevated triglycerides**						
No	45/513,914	Ref	Ref	55/969,624	Ref	Ref
Yes	121/824,315	1.20 (0.85–1.70)	0.87 (0.60–1.27)	41/614,577	0.76 (0.49–1.16)	0.70 (0.43–1.14)
**Low HDL cholesterol**						
No	110/978,365	Ref	Ref	64/1,117,811	Ref	Ref
Yes	47/258,324	1.25 (0.87–1.79)	1.02 (0.70–1.48)	24/310,035	1.19 (0.73–1.95)	1.22 (0.72–2.05)
**Hyperglycemia**						
No	104/1,189,508	Ref	Ref	79/1,467,713	Ref	Ref
Pre-diabetes	30/145,967	1.82 (1.21–2.74)^**^	1.70 (1.22–2.59)^*^	12/148,871	1.25 (0.68–2.30)	1.28 (0.69–2.39)
Diabetes	41/94,316	2.91 (2.00–4.23)^***^	2.88 (1.91–4.34)^***^	10/70,033	1.93 (1.01–3.79)^*^	2.43 (1.20–4.91)^*^

*Abbreviations*: *CI* confidence interval, *HDL* high density lipoproteins, *HR* hazard ratio, *MetS* metabolic syndrome

* 0.01 ≤ *P*-value < 0.05, ** 0.001 ≤ *P*-value < 0.01, ***P-value < 0.001

^a^Model 1: Multivariable adjusted Cox proportional hazards model adjusted for age in years, ethnic, education, index of multiple deprivation (a measure of socio-economic status), alcohol consumption, smoking status, physical activity, portions of fruit and vegetable intake, comorbidities, family history of cancer, menopause status (for the female only) and hormone replacement therapy (for the female only)

^b^Model 2: additionally adjusted for levels of individual component of MetS

**Fig. 1:**
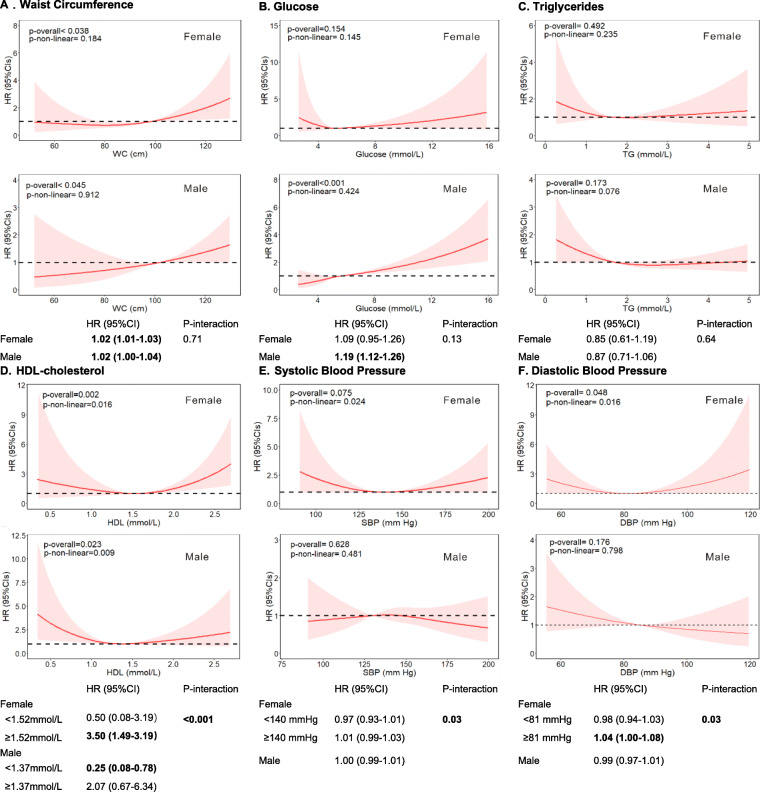
Associations between individual MetS components and risk of liver cancer, allowing for non-linear effects. A. Waist Circumference; B. Glucose; C. Triglycerides; D. High Density Lipoprotein; E. Systolic Blood Pressure; F. Diastolic Blood Pressure. The reference levels of MetS components in these plots (with HR fixed as 1.0) were 90 cm for waist circumference, 1.7 mmol/L for triglyceride, 1.52 mmol/L for HDL-cholesterol, 5.6 mmol/L for fasting glucose, 140.33 mmHg for systolic blood pressure and 81.29 mmHg for diastolic blood pressure. Separate models were fitted using restricted cubic spline with 4 knots at 5th, 35th, 65th, and 95th percentiles for each MetS component, adjusted for age at baseline, gender, education, ethnic, index of multiple deprivation, alcohol consumption, smoking status, physical activity, fruit and vegetable intake, comorbidities, family history of cancer, menopause status (for the female only) and hormone replacement therapy (for the female only) and levels of other MetS component. The “p-overall” represented the overall significance (including linear effect and nonlinear effect) for each MetS component and was derived using a likelihood ratio test comparing the model with linear and cubic spline terms of each MetS component against the model including only all covariates. The “p-non-linear” represented the nonlinear significance of nonlinear effect for each MetS component, and was derived using a likelihood ratio test comparing the model with linear and cubic spline terms of each MetS component against the model with only a linear term of each MetS component, after adjustment all covariates. Estimated HRs per unit derived from best fitting piecewise linear model. For the MetS components (HDL, SBP/DBP) showing U-associations, estimated HRs per unit were performed in two parts according to the point with the lowest risk. Abbreviation: CI, confidence interval; DBP, diastolic blood pressure; HDL, high density lipoprotein; HR, hazard ratio; SBP, systolic blood pressure; TG, triglycerides; WC, waist circumference

We further evaluated the non-linear associations of continuous individual MetS components and liver cancer risk by gender (Fig. 1). Higher WC and blood glucose were associated with substantially increased risk of liver cancer in both genders, with no evidence against non-linearity. Specifically, each centimeter increase in WC was associated with 2% increased risk for liver cancer in both sexes; The glucose was associated with an increased liver cancer risk in men (HR per 1 unit increase =1.19, 95% CI, 1.12–1.26), but not in women (HR per unit increase =1.09, 95% CI, 0.95–1.26). A U-shaped relationship between HDL cholesterol and liver cancer risk was observed, with an interaction effect by gender (p-interaction < 0.001). In men, HDL cholesterol was negatively associated with risk of liver cancer at the lower end of the HDL cholesterol range (< 1.37 mmol/L); whereas in women, the risk of liver cancer increased with increasing HDL cholesterol above 1.52 mmol/L. Gender also showed interaction effect in the associations between DBP/SBP and risk of liver cancer, with a U-shaped association shown only in women. Higher DBP was associated with substantial increase in risk if DBP was above 81.29 mmHg in women; whereas in men, there was likely to be a modest decrease in risk with no evidence against linearity. There was no sufficient evidence of association or interactive effect between TG and liver cancer risk by gender.

## Discussion

This prospective analysis of over 0.47 million individuals suggested that MetS, particularly for central obesity and hyperglycaemia, was associated with elevated risk of liver cancer in male participants, but not in females. For individual MetS components, WC and blood glucose were associated with increased liver cancer risk in a dose-dependent manner, while HDL cholesterol showed a U-shaped relationship. The associations of blood pressure and liver cancer risk were modified by gender, with a U-shaped association in women while no association in men. This study highlighted the importance of individualized liver cancer prevention by gender, especially in patients with MetS.

Accumulating literature demonstrated that MetS was associated with increased risk of liver cancer [5–8]. Our results were in agreement with a recent meta-analysis of six cohort studies, which showed that the presence of MetS was associated with higher incidence of HCC in male participants but not in females [8]. The gender-specific effect could be explained as different components of MetS may confer diverse risk of liver cancer by gender. In the present study, central obesity was associated with increased risk of liver cancer only in men but not in women. This finding was in agreement with two meta-analyses that suggested that sex disparity existed in the obesity and HCC association, with higher risk of HCC in obese men than in obese women [16, 17]. One explanation for this is that gender dimorphisms may occur in adipose homeostasis. Differences in body fat composition rather than BMI were suggested to be true determinants of liver cancer prognosis [18]. Visceral fat, which played a more important role in liver carcinogenesis than total adiposity accounts for a strikingly larger proportion of body fat in men than in women [19, 20]. Previous study has indicated that the body composition difference may be driven by differing sex hormones: higher androgen receptor density increased visceral fat and estrogen promoted the accumulation of subcutaneous fat, which protects against inflammation [21–24]. Evidence has also suggested the regulatory roles of the estrogen and/or androgen receptors signaling in the key metabolic microRNAs and chromatin-modifying enzymes in the pathogenesis of both type 2 diabetes and HCC [25]. This may explain why pre-diabetes affected liver cancer risk in men, as also reported by Chen et al. [26] Future studies are needed to confirm these findings and further investigate the underlying mechanisms.

With limited evidence concerning the potential contribution of dyslipidemia to the development of liver cancer, our data suggested that either very low or high levels of HDL cholesterol might increase the risk of liver cancer. Liver is a major organ of cholesterol metabolism. A previous study suggested that excess cholesterol intake increased the incidence of cirrhosis, which in turn, increased liver cancer risk in general population [27]. This is in agreement with the beneficial inhibitory effect of statins on liver cancer incidence [28, 29]. However, cholesterol was also reported negatively associated with the risk of liver cancer [30, 31]; Another prospective cohort of 27,724 participants from Japan found a positive association between low levels of HDL cholesterol and HCC [32]. Our finding on HDL cholesterol provided new insights to why dyslipidemia might have opposite effect on liver cancer incidence in aforementioned studies, and further suggested that controlling HDL cholesterol in an appropriate range by gender might be an effective primary prevention for liver cancer.

Hypertension has been reported to either with or without an increased liver cancer risk [30, 31]. In the present study, we did not identify sufficient evidence of association between hypertension and risk of liver cancer, which could be explained by the U-shaped association, especially in female participants. We did not identify sufficient evidence of association between hypertension and risk of liver cancer, which could be explained by the U-shaped association between blood pressure and liver cancer risk, especially in female participants. The mechanisms underlying the association between blood pressure and liver cancer risk remains unknown, although previous study has suggested that hepatocellular mitogenic effect of several hormones which are known to play a role in the development of hypertension [33]. Further studies are needed to clarify the potential mechanisms.

Hyperglycemia may play a role in the pathogenesis of liver cancer. The dose-dependent increase in the risk of liver cancer by blood glucose in our study was supported by several cohort or case–control studies [7, 34]. Indeed, participants with chronic hyperglycemia related to prolonged diabetes had higher risk for developing primary liver cancer [34]. Some medications against diabetes (for example, metformin), on the other side, may be preventative against liver cancer [35]. Several biological mechanisms have been proposed to explain the linkage. First, accelerated glucose metabolism may serve as an energetic supply for cancer cells, thus favoring tumor growth and metabolism [36]. In addition, hyperglycemia might promote hepatocarcinogenesis via stimulation of the insulin-like growth factor pathway, which was known to promote cell proliferation, inhibit apoptosis, and regulate differentiation, invasion and angiogenesis in tumorigenesis [37].

Our findings, consistent with most previous studies, suggested that central obesity was associated with increased risk of liver cancer [30, 38, 39]. Additionally, we found a linear association between WC and liver cancer even after adjusting for other MetS components, which was identical to other study that demonstrated a linear and positive association with liver cancer for BMI [39]. Considering the evidence that WC was strongly correlated with total body fat, especially among elderly individuals [40], our results supported the hypothesis that the development of liver cancer in obese individuals might be mediated through the development of NAFLD and non-alcoholic NASH [41]. Additionally, central obesity is associated with increased adipokines, insulin and reduced levels of adiponectin, which in turn, may increase the formation of reactive oxygen species and facilitate cellular lipid peroxidation and DNA oxidative damage, promoting hepatocarcinogenesis [42].

A key strength in our study is that, based on a nationwide prospective UK Biobank database with up to 9.6 years of follow-up and detailed measurements, we evaluated the gender-specific non-linear associations between individual components of MetS and the liver cancer risk, which was seldom reported in previous epidemiological studies. The observed U-shaped relationship for HDL cholesterol might provide novel insights underlying the pathological changes for liver cancer development. Furthermore, in the UK Biobank, cases were identified at their first diagnosis by cancer registry, therefore information of outcome was verified. Details of histological type of liver cancer were also provided allowing us to include all reported HCC. While alcohol use was based on touchscreen questionnaire, the information was verified by a trained nurse. More importantly, the availability of a wide range of known and putative risk factor data from UK Biobank allowed us to adequately control other potential confounding effects.

Our study had several limitations. First, as an observational study, the causality between MetS and development of liver cancer could not be confirmed, although we capitalized on complimentary analytic methods to robustly assess their epidemiological relationship. Second, although various covariates were controlled in the models, residual confounding effects could not be excluded particularly for hepatitis virus infection and the status of underlying chronic liver disease. However, it is controversial that hepatitis viral infection is related to metabolic risk factors and studies assessing the association between metabolic comorbidities and liver cancer showed no major changes in risk after excluding individuals with HBV or HCV [7, 43]. In the present study, the primary results were also not altered after exclusion of individuals with either hepatitis or liver failure/cirrhosis. Third, in the case of diabetes, liver cancer risk might vary depending on the glucose control status and the disease duration in patients with diabetes. However, our study was unable to assessment of the associations as these information were lacking. Future prospective cohort studies with detailed information on diabetes are needed to investigate the potential effects. Fourth, with limited cases, this study was underpowered to estimate the association between MetS factors and liver cancer by different ages and alcohol use status. Future prospective studies on this field are required to assess these effects in details. Finally, considering UK Biobank participants differed from the general UK population with regard to a range of sociodemographic and health-related characteristics, the possible “healthy volunteer” effect in the UK Biobank might limit the generalization of our findings. However, its large size and the heterogeneity of exposure measures might provide valid scientific inferences of exposure-outcome relationship that are generalizable to other populations [44].

Overall, this large prospective study suggested a gender-specific linear or U-shaped associations between individual MetS components and the risk of liver cancer. The males with MetS, particularly central obesity and hyperglycaemia, were associated with increased risk of liver cancer. Although the casual relationship has not yet been confirmed, approaches to control the recent worldwide epidemic of MetS might benefit for a reduction in the liver cancer burden. Additionally, since the HDL cholesterol in both genders, and blood pressure in females presented U-shaped associations with liver cancer, it’s important to consider the extra liver cancer risk in individuals with high HDL cholesterol and low blood pressure, which are generally regarded as beneficial for cardiovascular health. Controlling the levels of these health indicators in an appropriate range might be an effective primary prevention to decrease liver cancer risk.

## Supplementary Information

**Additional file 1: Figure S1.** Flowchart of the participant’s enrollments. **Table S1.** Gender-specific hazard ratios and 95% confidence intervals for liver cancer by metabolic syndrome. **Table S2.** Sensitivity analyses of metabolic syndrome and risk of liver cancer.

## Data Availability

The data that support the findings of this study are available from UK Biobank (application number 51671, approved August 2019) but restrictions apply to the availability of these data, which were used under license for the current study, and so are not publicly available. Data are however available from the authors upon reasonable request and with permission of UK Biobank. UK Biobank is an open access resource, and the study website https://www.ukbiobank.ac.uk/ has information on available data and access procedures. Data sets used for the analysis will be made available under reasonable requests. All participants had provided written informed consent prior to data collection. Details on the study design and assessments of the UK Biobank cohort have been published elsewhere [14].
