# Supplementary material for: Metabolic syndrome and its component traits present gender-specific association with liver cancer risk: a prospective cohort study
Source: BMC Cancer. 2021 Oct 7;21:1084. doi: 10.1186/s12885-021-08760-1 (PMC8499577; doi:10.1186/s12885-021-08760-1)
Supplement: Supplementary file 1 — Additional file 1: Figure S1. Flowchart of the participant’s enrollments. Table S1. Gender-specific hazard ratios and 95% confidence intervals for liver cancer by metabolic syndrome. Table S2. Sensitivity analyses of metabolic syndrome and risk of liver cancer. [file 12885_2021_8760_MOESM1_ESM.docx]

**Metabolic Syndrome and its Component Traits Present Gender-specific Association with Liver Cancer Risk: a Prospective Cohort Study**

**Bin Xia ^1,2,3,^ Jianjun Peng ^4^, De Toni Enrico ^5^, Kuiqing Lu ^1^, Eddie C. Cheung ^3,6^, Zichong Kuo ^3^, Qiangsheng He ^1,2^, Yan Tang ^1^, Anran Liu ^7^, Die Fan ^1^, Changhua Zhang ^1,3^, Yihang Pan ^2,8^,Yulong He ^2,3*^, Jinqiu Yuan ^1,2,3*^, Shuo Fang ^9*^**

1. Clinical Research Center, The Seventh Affiliated Hospital, Sun Yat-sen University, Shenzhen, Guangdong, 518107, China;

2. Big Data Centre, The Seventh Affiliated Hospital, Sun Yat-sen University, Shenzhen, Guangdong, 518107, China

3. Guangdong Provincial Key Laboratory of Gastroenterology, Center for Digestive Disease, The Seventh Affiliated Hospital, Sun Yat-sen University, Shenzhen, Guangdong, China

4. Department of Gastrointestinal Surgery, The First Affiliated Hospital of Sun Yat-sen University, Guangzhou, Guangdong, China

5. Department of Medicine II, University Hospital, LMU Munich, Munich, Germany

6. Division of Gastroenterology, Davis School of Medicine, University of California, Oakland, USA

7. Department of Clinical Nutrition, The Seventh Affiliated Hospital, Sun Yat-sen University, Shenzhen, Guangdong, China

8. Precision Medicine Center, Scientific Research Center, The Seventh Affiliated Hospital, Sun Yat-sen University, Shenzhen, Guangdong, China

9. Department of Oncology, The Seventh Affiliated Hospital, Sun Yat-sen University, Shenzhen, Guangdong, 518107, China

*** Correspondence:**Yulong He
[heyulong@mail.sysu.edu.cn](mailto:heyulong@mail.sysu.edu.cn)

Jin-Qiu Yuan
[yuanjq5@mail.sysu.edu.cn](mailto:yuanjq5@mail.sysu.edu.cn)

Shuo Fang

fangsh9@mail.sysu.edu.cn

SUPPLEMENTARY APPENDIX

**Figure S1.** Flowchart of the participant's enrollments.

**Table S1.** Gender-specific hazard ratios and 95% confidence intervals for liver cancer by metabolic syndrome.

**Table S2.** Sensitivity analyses of metabolic syndrome and risk of liver cancer.


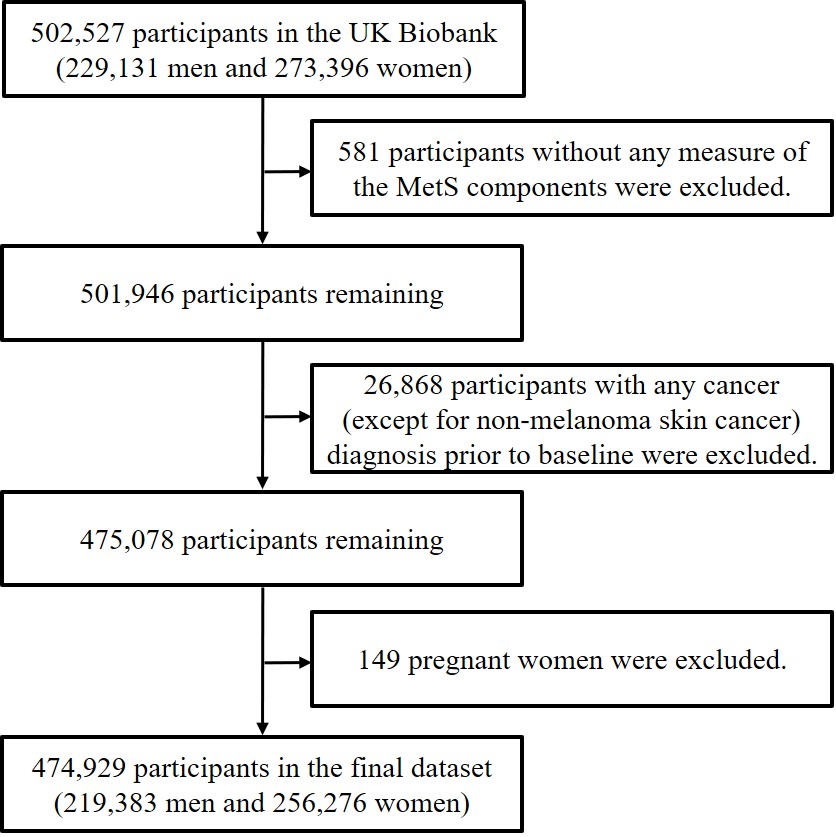


**Figure S1.** Flowchart of the participant's enrollments.

**Table S1.** Gender-specific hazard ratios and 95% confidence intervals for liver cancer risk by different metabolic syndrome definition criteria.

|  | **Male** | |  | **Female** | |
| --- | --- | --- | --- | --- | --- |
|  | **No. of cases/ Person-years** | **HR (95% CI) ^a^** |  | **No. of cases/ Person-years** | **HR (95% CI) ^a^** |
| **Presence of MetS according to IDF criteria** | | | | | |
| No | 93/1060709 | Ref |  | 76/1308130 | Ref |
| Yes | 82/366987 | **1.81 (1.34-2.46) ***** |  | 25/376740 | 0.77 (0.48-1.23) |
| **Presence of MetS according to NCEP ATP III criteria** | | | | | |
| No | 99/1084089 | Ref |  | 75/1351428 | Ref |
| Yes | 76/345686 | **1.85 (1.36-2.51) ***** |  | 26/335195 | 1.00 (0.63-1.58) |
| **Presence of MetS according to AHA/NHLBI criteria** | | | | | |
| No | 68/956135 | Ref |  | 74/1249763 | Ref |
| Yes | 107/473640 | **2.13 (1.56-2.92) ***** |  | 27/436860 | 0.68 (043-1.07) |

^***^ *P*-value < 0.001

^a^ Estimates were assessed by multivariable adjusted Cox proportional hazards model adjusted for age in years, ethnic, education, index of multiple deprivation (a measure of socio-economic status), alcohol consumption, smoking status, physical activity, portions of fruit and vegetable intake, comorbidities, family history of cancer, menopause status (for the female only) and hormone replacement therapy (for the female only).

Abbreviations: AHA/NHLBI, american heart association/national heart, lung and blood Institute; CI, confidence interval; HR, hazard ratio; IDF, international diabetes federation; NCEP ATP III, national cholesterol education program adult treatment panel III; MetS, metabolic syndrome.

**Table S2.** Sensitivity analyses of metabolic syndrome and risk of liver cancer

|  | **Male** | |  | **Female** | |
| --- | --- | --- | --- | --- | --- |
|  | **No of cases/  Person-years** | **HR (95% CI) ^a^** |  | **No of cases/  Person-years** | **HR (95% CI) ^a^** |
| Lagging the exposure for 2 years and allow a time window for liver cancer risk. | | | | | |
| Without MetS | 70/1057557 | Ref |  | 58/1305422 | Ref |
| With MetS | 69/365659 | **2.04 (1.45,2.88) ^***^** |  | 19/375642 | 0.75 (0.44,1.27) |
| Excluding individuals with history of hepatitis, liver failure or cirrhosis | | | | | |
| Without MetS | 87/1058782 | Ref |  | 70/1306131 | Ref |
| With MetS | 74/366155 | **1.93 (1.41,2.66) ^***^** |  | 22/376065 | 0.76 (0.47,1.25) |
| Complete-case analysis | | | | | |
| Without MetS | 49/646139 | Ref |  | 39/749738 | Ref |
| With MetS | 35/201786 | **1.74 (1.12,2.72) ^*^** |  | 11/180027 | 0.90 (0.45,1.79) |

^*^ 0.01≤ *P*-value < 0.05, ^***^ *P*-value < 0.001

^a^ Estimates were assessed by multivariable adjusted Cox proportional hazards model adjusted for age in years, ethnic, education, index of multiple deprivation (a measure of socio-economic status), alcohol consumption, smoking status, physical activity, portions of fruit and vegetable intake, comorbidities, family history of cancer, menopause status (for the female only) and hormone replacement therapy (for the female only).

Abbreviation: CI, confidence interval; HR, hazard ratio; MetS, metabolic syndrome.
